# Using Machine Learning to Identify Features from Home Sensors that Predict Care Partner Burden

**DOI:** 10.1002/alz70858_107291

**Published:** 2025-12-26

**Authors:** Neil W. Thomas, Julien Larivière‐Chartier, Bahareh Chimehi, Rajib Dey, Laura Ault, Bruce Wallace, Frank Knoefel, Jeffrey A Kaye, Zachary T Beattie, Lisa Sheehy, Joel S. Steele, Lyndsey M. Miller

**Affiliations:** ^1^ AGE‐WELL NIH SAM3, Ottawa, ON, Canada; ^2^ University of Ottawa, Ottawa, ON, Canada; ^3^ Bruyere Health Research Institute, Ottawa, ON, Canada; ^4^ Carleton University, Ottawa, ON, Canada; ^5^ Oregon Health & Science University, Portland, OR, USA; ^6^ NIA‐Layton Aging & Alzheimer's Disease Center, Portland, OR, USA; ^7^ Oregon Center for Aging & Technology (ORCATECH), Portland, OR, USA; ^8^ Portland Veterans Affairs Health Care System, Portland, OR, USA; ^9^ University of North Dakota, Grand Forks, ND, USA

## Abstract

**Background:**

Assessing care‐related activities and burden most often involves self‐report, without objective information on activities in the home. Previous work has demonstrated that home sensor data from daily activities can be collected over extended time periods and is acceptable, though it is unknown which activities in the home contribute to care partner burden. This project aims to develop a digital signature that identifies the level of care partner burden associated with PLWD/care partner dyads living in the community.

**Method:**

Clinical and sensor data from longitudinal studies involving the Oregon Center for Aging & Technology platform were analyzed using machine learning (ML). Participant dyads consisted of a care partner living with an individual with mild cognitive impairment (MCI) or dementia. Care partners completed the Zarit Burden Interview Short Form (ZBI‐12) weekly for up to 18 months. Data analyzed from motion sensors placed in each room in a participant's home are presented here. Features from the sensor data (e.g., time spend in different rooms in the home, number of trips to each room) were evaluated with independent component analysis (ICA) and a decision tree ML model. SHAP values representing how much each feature contributed to the model were generated to identify the most relevant prediction of low versus high burden.

**Result:**

Data from 44 dyads contributed to the model. Using only data from the motion sensor, the model's accuracy was 69.8% to predict low versus high burden. ICA generated 3 components with a mix of sensor data features. SHAP value analysis identified one component that included features that more distinctly corresponded to low or high burden based on ZBI‐12 scores. Features that more strongly contributed to high levels of burden included the number of trips to the bathroom and average time spend in the bathroom.

**Conclusion:**

Home sensors may provide a method to continuously assess daily activities that contribute to the stress level and burden of care partners. Novel ML analysis techniques could help to identify relevant outcome measures from the large amount of data collected by home sensors. Ongoing work is determining the combination of sensors from the technology platform that best predict burden level.

